# As Old as the Hills: Montane Scorpions in Southwestern North America Reveal Ancient Associations between Biotic Diversification and Landscape History

**DOI:** 10.1371/journal.pone.0052822

**Published:** 2013-01-09

**Authors:** Robert W. Bryson, Brett R. Riddle, Matthew R. Graham, Brian Tilston Smith, Lorenzo Prendini

**Affiliations:** 1 School of Life Sciences, University of Nevada, Las Vegas, Las Vegas, Nevada, United States of America; 2 Department of Biology and Burke Museum of Natural History and Culture, University of Washington, Seattle, Washington, United States of America; 3 Museum of Natural Science, Louisiana State University, Baton Rouge, Louisiana, United States of America; 4 Division of Invertebrate Zoology, American Museum of Natural History, New York, New York, United States of America; Onderstepoort Veterinary Institute, South Africa

## Abstract

**Background:**

The age of lineages has become a fundamental datum in studies exploring the interaction between geological transformation and biotic diversification. However, phylogeographical studies are often biased towards lineages that are younger than the geological features of the landscapes they inhabit. A temporally deeper historical biogeography framework may be required to address episodes of biotic diversification associated with geologically older landscape changes. Signatures of such associations may be retained in the genomes of ecologically specialized (stenotopic) taxa with limited vagility. In the study presented here, genetic data from montane scorpions in the *Vaejovis vorhiesi* group, restricted to humid rocky habitats in mountains across southwestern North America, were used to explore the relationship between scorpion diversification and regional geological history.

**Results:**

Strong phylogeographical signal was evident within the *vorhiesi* group, with 27 geographically cohesive lineages inferred from a mitochondrial phylogeny. A time-calibrated multilocus species tree revealed a pattern of Miocene and Pliocene (the Neogene period) lineage diversification. An estimated 21 out of 26 cladogenetic events probably occurred prior to the onset of the Pleistocene, 2.6 million years ago. The best-fit density-dependent model suggested diversification rate in the *vorhiesi* group gradually decreased through time.

**Conclusions:**

Scorpions of the *vorhiesi* group have had a long history in the highlands of southwestern North America. Diversification among these stenotopic scorpions appears to have occurred almost entirely within the Neogene period, and is temporally consistent with the dynamic geological history of the Basin and Range, and Colorado Plateau physiographical provinces. The persistence of separate lineages at small spatial scales suggests that a combination of ecological stenotopy and limited vagility may make these scorpions particularly valuable indicators of geomorphological evolution.

## Introduction

The strength of the interplay between geological transformation and biotic diversification—geobiotic history [1]—is an old and contentious issue in biology [2]. With the advent of molecular-based estimates of lineage relationships and ages, traditional dichotomies between views that posit either strong adherence to ‘Earth and life evolving together’ or ‘life diversifying largely without dependence on Earth history events’ have been tempered by studies showing that species assemblages often exhibit a temporally wide range of idiosyncratic responses to Earth history events [3], [4], [5]. Although the age of lineages has become a fundamental datum for investigating relationships between Earth and biotic history, studies of diversification within the popular phylogeographical [6] paradigm have been biased towards relatively young lineages [7], [8]. Expansion of the temporal context of a study beyond the relatively recent timeframes encompassing phylogeography could capture a record of biotic diversification associated with geologically older landscape changes. Such changes may be retained in the genomes of ecologically specialized (stenotopic) taxa with limited vagility.

Owing to their antiquity, morphological, and ecological conservatism, scorpions are excellent candidates for investigating such deep geobiotic histories [9]. Derived from amphibious ancestors that lived more than 425 million years ago (Ma), scorpions represent an ancient radiation of terrestrial arthropods, earning the title ‘living fossils’ [10]. Their conserved morphology implies relative stasis in ecological requirements over time [9]. Many scorpions demonstrate limited vagility and a high degree of stenotopy such that the distributions of phylogenetically related taxa are often predictably restricted to a narrow range of stable habitats that may have persisted over millions of years [9], [11]. As evidence, several evolutionary studies on scorpions have revealed patterns of deep species-level divergence and diversification during the Miocene and Pliocene [12], [13], [14], [15], [16]. The diversity and ecological specialization of North American scorpions [17], [18] makes them appropriate for exploring biogeographical patterns on the continent.

**Figure 1:**
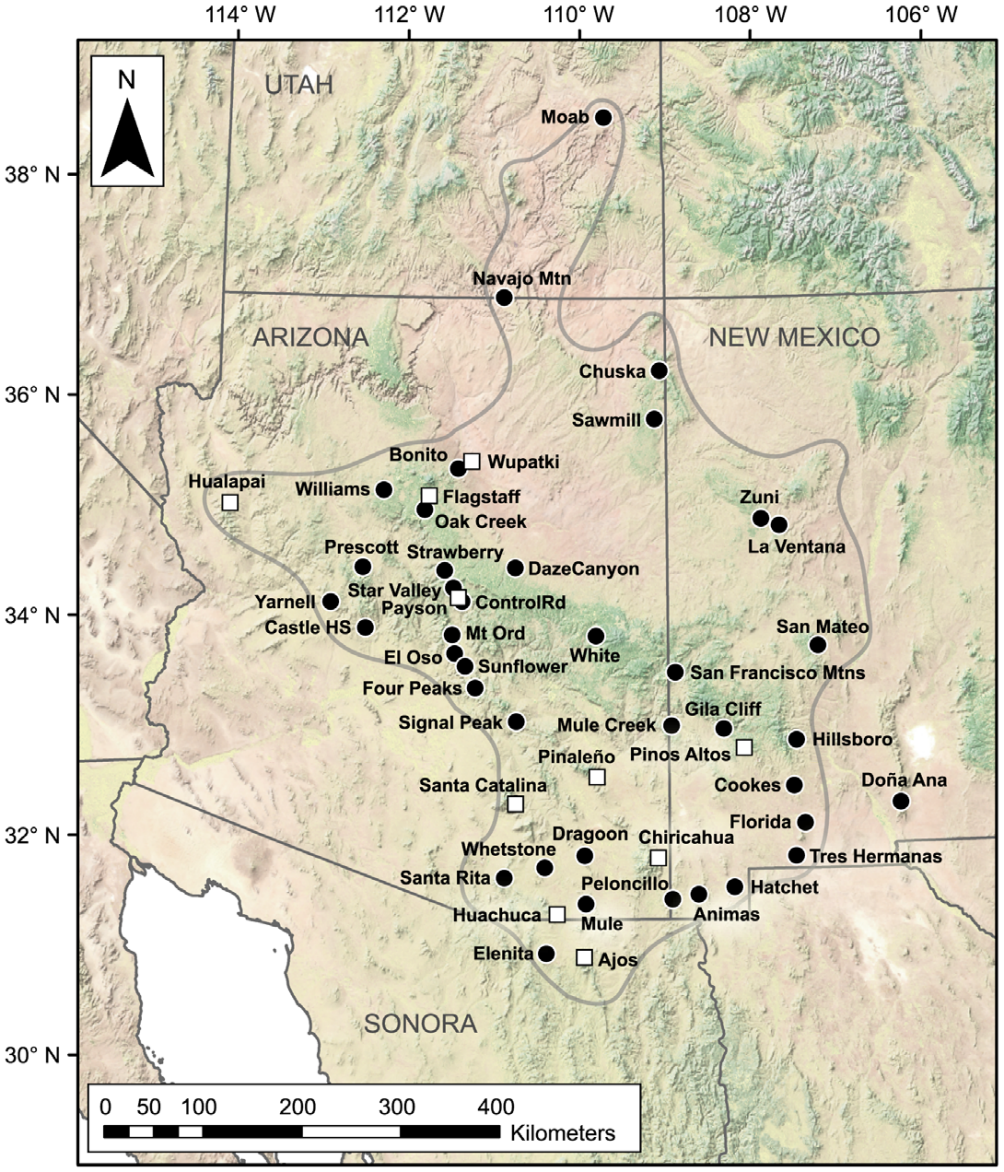
Collection localities for genetic samples of scorpions in the *Vaejovis vorhiesi* group included in this study. White squares indicate type localities of described species (see Table 1), and grey line indicates approximate known distribution of the group.

In the present study, the historical diversification of montane scorpions in the *Vaejovis vorhiesi* group (Vaejovidae) was examined as part of a comparative approach aimed at reconstructing the paleohistory of highlands in southwestern North America [19], [20]. The *vorhiesi* group, comprising 12 described and several undescribed species, appears to be restricted to humid, rocky habitats in mixed pine-oak-juniper woodlands [21], [22] across their known distribution from central Utah southwards through Arizona and New Mexico to Sonora in northern Mexico (Fig. 1). Diversification in these scorpions appears to be constrained by habitat specialization, and divergence may have been promoted by allopatric speciation in isolated rocky highland habitats, as proposed for scorpion taxa in other parts of the world [9], [11]. If ephemeral woodland corridors that developed during Pleistocene glacial periods and connected highland biota [20], [23] lacked suitable humid, rocky habitats, dispersal between adjacent mountain ranges inhabited by stenotopic scorpions in the *vorhiesi* group, would be limited.

Based on their relatively specialized ecological requirements for humid, rocky habitats and therefore close association with geological features, montane scorpions of the *vorhiesi* group may show signatures of pre-Pleistocene divergence, contrary to the phylogeographical patterns observed in many co-distributed taxa [23], [24], [25], [26]. These scorpions may thus offer novel insights into the deeper geobiotic history of the highland landscapes of southwestern North America. A mitochondrial DNA (mtDNA) dataset was generated from 63 samples of *vorhiesi* group scorpions and their relatives to explore phylogeographical structure, and a species tree reconstructed using multilocus data (mtDNA and two nuclear genes). Divergence times were estimated across the mtDNA dataset and the multilocus species tree, and the temporal distribution of divergence events was modeled across southwestern North America. These reconstructions and models were used to explore the relationship between diversification and regional geological history. Diversification within the *vorhiesi* group appears to have occurred almost entirely within the Neogene period, and may be related to the dynamic geological history of southwestern North America.

## Methods

### Ethics statements

Fieldwork in Mexico was conducted under permits granted by the Secretaría de Medio Ambiente y Recursos Naturales (SEMARNAT) to O.F. Francke, the late F. Mendoza-Quijano, and C. Solis-Rojas. The Navajo Nation and National Parks Service granted permits for collection in northern Arizona and New Mexico.

### Genetic data

DNA sequence data were generated from 63 samples of *vorhiesi* group scorpions collected from throughout the known range (Fig. 1, Table 1). All described species in the group (*V. bandido*, *V. cashi*, *V. crumpi*, *V. deboerae*, *V. electrum*, *V. feti*, *V. halli*, *V. jonesi*, *V. lapidicola*, *V. paysonensis*, *V. vorhiesi*) were represented with the exception of *V. bigelowi*. The sister taxon to the group is uncertain, so additional samples from 10 other geographically proximate montane *Vaejovis* were included. *Paruroctonus boreus* was used as the outgroup [27].

**Table 1:** Collection data for genetic samples of scorpions in the *Vaejovis vorhiesi* group.

Sample ID	Taxon	Locality
**Ajos SON**	*Vaejovis bandido*	Mexico: Sonora: Sierra de los Ajos
**Chiricahua AZ**	*Vaejovis cashi*	USA: Arizona: Chiricahua Mountains*
Chiricahua 2 AZ	*Vaejovis cashi*	USA: Arizona: Chiricahua Mountains*
Chiricahua 3 AZ	*Vaejovis cashi*	USA: Arizona: Chiricahua Mountains*
**Prescott AZ**	*Vaejovis crumpi*	USA: Arizona: Prescott*
**Prescott 2 AZ**	*Vaejovis crumpi*	USA: Arizona: Prescott*
**Santa Catalina AZ**	*Vaejovis deboerae*	USA: Arizona: Santa Catalina Mountains*
**Pinaleño AZ**	*Vaejovis electrum*	USA: Arizona: Pinaleño Mountains*
**Pinaleño 2 AZ**	*Vaejovis electrum*	USA: Arizona: Pinaleño Mountains*
Pinaleño 3 AZ	*Vaejovis electrum*	USA: Arizona: Pinaleño Mountains*
**Pinos Altos NM**	*Vaejovis feti*	USA: New Mexico: Pinos Altos*
**Mt Ord AZ**	*Vaejovis halli*	USA: Arizona: Mazatzal Mountains, Mount Ord*
Mt Ord 2 AZ	*Vaejovis halli*	USA: Arizona: Mazaztal Mountains, Mount Ord*
**Wupatki AZ** ^IT^	*Vaejovis jonesi*	USA: Arizona: Wupatki National Monument*
**Flagstaff AZ** ^IT^	*Vaejovis lapidicola*	USA: Arizona: Flagstaff*
**Payson AZ** ^IT^	*Vaejovis paysonensis*	USA: Arizona: Payson*
**Hualapai AZ**	*Vaejovis tenuipalpus*	USA: Arizona: Hualapai Mountains*
**Hualapai 2 AZ**	*Vaejovis tenuipalpus*	USA: Arizona: Hualapai Mountains*
**Huachuca AZ**	*Vaejovis vorhiesi*	USA: Arizona: Huachuca Mountains*
Huachuca 2 AZ	*Vaejovis vorhiesi*	USA: Arizona: Huachuca Mountains*
**Animas NM**	*Vaejovis* sp., *vorhiesi* group	USA: New Mexico: Animas Mountains
**Bonito AZ**	*Vaejovis* sp., *vorhiesi* group	USA: Arizona: NE Flagstaff, near Bonito
**Castle HS AZ**	*Vaejovis* sp., *vorhiesi* group	USA: Arizona: Castle Hot Springs
**Chuska AZ**	*Vaejovis* sp., *vorhiesi* group	USA: Arizona: Chuska Mountains
Chuska 2 AZ	*Vaejovis* sp., *vorhiesi* group	USA: Arizona: Chuska Mountains
**Control Rd AZ**	*Vaejovis* sp., *vorhiesi* group	USA: Arizona: S side of Control Road
**Cookes NM**	*Vaejovis* sp., *vorhiesi* group	USA: New Mexico: Cooke's Range
**Cookes 2 NM**	*Vaejovis* sp., *vorhiesi* group	USA: New Mexico: Cooke's Range
**Daze Canyon AZ**	*Vaejovis* sp., *vorhiesi* group	USA: Arizona: Daze Canyon
**Doña Ana NM**	*Vaejovis* sp., *vorhiesi* group	USA: New Mexico: Aguirre Springs National Recreation Area
**Dragoon AZ**	*Vaejovis* sp., *vorhiesi* group	USA: Arizona: Dragoon Mountains
**El Oso AZ**	*Vaejovis* sp., *vorhiesi* group	USA: Arizona: Mazatzal Mountains, El Oso Mine
**Elenita SON**	*Vaejovis* sp., *vorhiesi* group	Mexico: Sonora: Sierra Elenita
**Florida Mtns NM**	*Vaejovis* sp., *vorhiesi* group	USA: New Mexico: Little Florida Mountains
Four Peaks AZ	*Vaejovis* sp., *vorhiesi* group	USA: Arizona: Four Peaks
**Gila Cliff NM**	*Vaejovis* sp., *vorhiesi* group	USA: New Mexico: nr. Gila Cliff Dwellings
**Hatchet NM**	*Vaejovis* sp., *vorhiesi* group	USA: New Mexico: Big Hatchet Mountains
Hillsboro NM	*Vaejovis* sp., *vorhiesi* group	USA: New Mexico: W Hillsboro
La Ventana NM	*Vaejovis* sp., *vorhiesi* group	USA: New Mexico: La Ventana Arch
**Moab UT**	*Vaejovis* sp., *vorhiesi* group	USA: Utah: Moab
**Mule Creek NM**	*Vaejovis* sp., *vorhiesi* group	USA: New Mexico: Mule Creek
**Mule Creek 2 NM**	*Vaejovis* sp., *vorhiesi* group	USA: New Mexico: Mule Creek
**Mule Mtns AZ**	*Vaejovis* sp., *vorhiesi* group	USA: Arizona: Mule Mountains
**Navajo Mtn UT**	*Vaejovis* sp., *vorhiesi* group	USA: Utah: Navajo Mountain
**Oak Creek AZ**	*Vaejovis* sp., *vorhiesi* group	USA: Arizona: Oak Creek, SW Flagstaff
Oak Creek 2 AZ	*Vaejovis* sp., *vorhiesi* group	USA: Arizona: Oak Creek, SW Flagstaff
Peloncillo 2 NM	*Vaejovis* sp., *vorhiesi* group	USA: New Mexico: Peloncillo Mountains
**Peloncillo NM**	*Vaejovis* sp., *vorhiesi* group	USA: New Mexico: Peloncillo Mountains
**San Francisco Mtns** NM	*Vaejovis* sp., *vorhiesi* group	USA: New Mexico: San Francisco Mountains, Blue Range Wilderness
**San Mateo NM**	*Vaejovis* sp., *vorhiesi* group	USA: New Mexico: San Mateo Mountains
**Santa Rita AZ**	*Vaejovis* sp., *vorhiesi* group	USA: Arizona: Santa Rita Mountains
**Santa Rita 2 AZ**	*Vaejovis* sp., *vorhiesi* group	USA: Arizona: Santa Rita Mountains
**Sawmill AZ**	*Vaejovis* sp., *vorhiesi* group	USA: Arizona: Sawmill
Signal Peak AZ	*Vaejovis* sp., *vorhiesi* group	USA: Arizona: Signal Peak
Star Valley AZ	*Vaejovis* sp., *vorhiesi* group	USA: Arizona: Star Valley nr. Payson
**Strawberry AZ**	*Vaejovis* sp., *vorhiesi* group	USA: Arizona: Strawberry
Sunflower AZ	*Vaejovis* sp., *vorhiesi* group	USA: Arizona: Sunflower
**Tres Hermanas NM**	*Vaejovis* sp., *vorhiesi* group	USA: New Mexico: Tres Hermanas Mountains
**Whetstone AZ** ^CO, IT^	*Vaejovis* sp., *vorhiesi* group	USA: Arizona: Whetstone Mountains
**White Mtns AZ**	*Vaejovis* sp., *vorhiesi* group	USA: Arizona: White Mountains near Pinetop
**Williams AZ**	*Vaejovis* sp., *vorhiesi* group	USA: Arizona: Williams
**Yarnell AZ**	*Vaejovis* sp., *vorhiesi* group	USA: Arizona: Yarnell
**Zuni NM**	*Vaejovis* sp., *vorhiesi* group	USA: New Mexico: Zuni Mountains
***V. franckei***	*Vaejovis franckei*	Mexico: Oaxaca: Cerro Corral del Piedra
***V. granulatus***	*Vaejovis granulatus*	Mexico: Morelos: W of Huitzilac
***V. montanus*** ** CHIH**	*Vaejovis montanus*	Mexico: Chihuahua: Zorillo*
*V. montanus* CHIH 2	*Vaejovis montanus*	Mexico: Chihuahua: El Vergel
***V. montanus*** ** SON**	*Vaejovis montanus*	Mexico: Sonora: Yécora
***V. nayarit***	*Vaejovis nayarit*	Mexico: Nayarit: Jesus María Corte*
***V. pattersoni***	*Vaejovis pattersoni*	Mexico: Baja California Sur: Sierra de la Laguna*
***Vaejovis*** ** sp. “Mezquital” DGO**	*Vaejovis* sp., *mexicanus* group	Mexico: Durango: Mezquital
***Vaejovis*** ** sp. “Mimbres” DGO**	*Vaejovis* sp., *mexicanus* group	Mexico: Durango: Hwy 40, Rio Mimbres
***Vaejovis*** ** sp. “del Nido”**	*Vaejovis* sp., *mexicanus* group	Mexico: Chihuahua: Sierra del Nido
***Paruroctonus boreus***	*Paruroctonus boreus*	USA: Nevada: North Fork, Humboldt River

All samples are deposited in the Ambrose Monell Cryocollection (AMCC) at the American Museum of Natural History, New York. Asterisks denote samples collected in the vicinity of the type localities. Samples in bold were used in multilocus phylogenetic estimates (letter abbreviations indicate samples with missing data: ^CO^, COI; ^IT^, ITS2). Sequences were deposited in GenBank (accession numbers JX909361–JX909616).

Fragments of mitochondrial DNA were sequenced from a protein-coding gene (cytochrome *c* oxidase I, COI; 854 base pairs, or bp) and a ribosomal gene (16S rDNA, 16S; 404 bp). Two nuclear genes were also sequenced for a subset of samples (*n* = 39) representing geographically cohesive lineages within the *vorhiesi* group: 526 bp of 28S rDNA (28S) and 319 bp of the internal transcribed spacer region (ITS2) between the 5.8S rDNA and 28S rDNA. Taxon-specific mtDNA primers (Table S1) were used to avoid co-amplification of nuclear mitochondrial pseudogenes (numts) [28], and chromatograms examined for the presence of double peaks [29], indels, frameshifts, and premature stop codons. Primer sequences for nuclear genes were taken from Tully et al. [30] for 28S and Ji et al. [31] for ITS2. Genomic DNA was extracted from leg muscle tissue, and lab protocols provided in Bryson et al. [19] and Prendini et al. [32], [33] were followed to generate sequence data. Complete genetic data could not be obtained for several samples (Table 1). Sequence alignments for individual gene regions were performed with MAFFT v6 [34], [35] using default settings, the ‘1PAM/k = 2’ scoring matrix for nucleotide sequences, and the Q-INS-i algorithm for 16S and 28S data and the E-INS-i algorithm for ITS2 data. Heterozygous sites in nuclear segments were identified when two different nucleotides were present at the same position in electropherograms of both strands, with the weaker peak reaching at least 50% of the stronger signal. The gametic phases of the variants were computationally determined using PHASE v2.1.1 [36]. Five separate runs of 400 iterations were conducted for each nuclear dataset, and results with a probability threshold of 0.7 or higher accepted. All polymorphic sites with a probability less than 0.7 in both alleles were coded with the appropriate IUPAC ambiguity code. A partitioned homogeneity test with 1000 heuristic replicates was performed in PAUP* v4.0b10 [37] to test for conflicting phylogenetic signals between the mitochondrial and nuclear genes.

### Phylogeographical estimation

The mtDNA dataset was analyzed to examine geographical structure and delineate geographically cohesive lineages within the *vorhiesi* group using Bayesian inference in MrBayes v3.1 [38]. Lineages were defined as genetically distinct geographical clusters with strong support values (≥0.95 Bayesian posterior probability), consistent with definitions of the term “phylogroup” [39], [40], [41]. Separate models were implemented for 16S and for each gene codon position of COI, the best partitioning scheme chosen based on Bayes Factors [42]. MrModeltest v2.1 [43] was used to select a best-fit model of nucleotide evolution, based on Akaike information criteria (AIC), for each partition. Bayesian settings included random starting trees, a variable rate prior, and heating temperature of 0.05. Analyses were run for 4 million generations, sampling every 100 generations, and output parameters visualized using the program TRACER v1.4 [44] to ascertain stationarity and whether the duplicated runs had converged on the same mean likelihood. All samples obtained during the first one million (25%) generations were discarded as burn-in, and a 50% majority-rule consensus phylogram with nodal posterior probability support estimated from the combination of the four runs post-burn-in.

### Species tree and divergence date estimation

**Figure 2:**
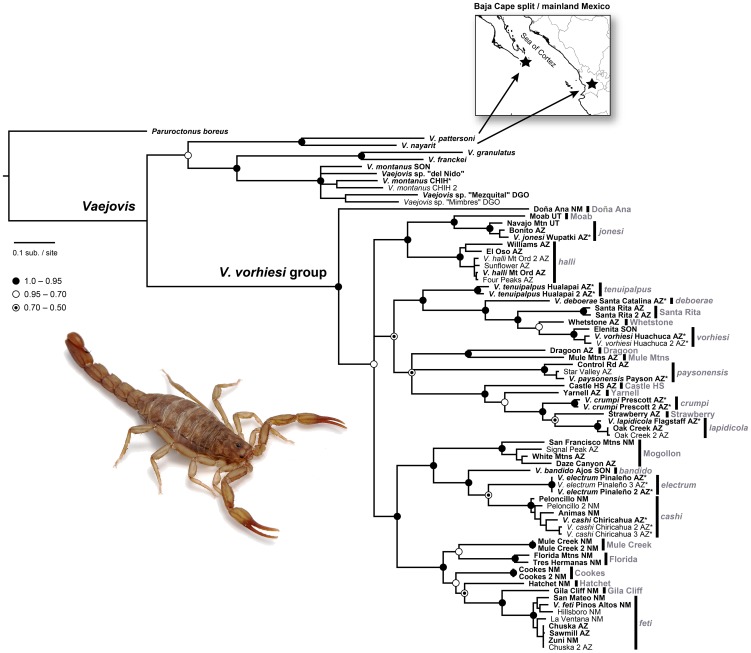
Mitochondrial phylogeny of scorpions in the *Vaejovis vorhiesi* group. Phylogeny inferred from Bayesian analyses of 1258 bp of concatenated COI and 16S mitochondrial sequence data. Posterior probability support values for nodes are indicated by coded dots explained in the figure legend. Inferred lineages are indicated by black bars. Samples used in species tree reconstruction are indicated in bold font. Localities of haplotypes are listed in Table 1. Inset depicts the geographical localities of putative sister species of montane *Vaejovis* isolated on opposite sides of the Sea of Cortez. The divergence of these two species caused by the split of the Cape region of Baja California from mainland Mexico was used to calibrate the molecular clock.

A species tree was reconstructed and divergence times within the *vorhiesi* group estimated from the multilocus dataset using *BEAST [45], a part of the BEAST v1.6.2 package. One to five exemplar samples were used from each geographical mtDNA lineage. Best-fit models of evolution were selected using MrModeltest, a Yule process speciation prior applied, and relaxed uncorrelated lognormal clocks specified for each gene tree. Trees were linked in analyses of the COI and 16S mtDNA data, which represent a single locus, and substitution and clock models were unlinked. The separation of the Cape region of Baja California from mainland Mexico between 7.5–14 Ma ([46], [47]; reviewed in [48]) was inferred as the causal mechanism for divergence between *V. pattersoni* and *V. nayarit*, putative sister species of montane *Vaejovis* isolated on opposite sides of the Sea of Cortez (Fig. 2). This split was used to calibrate the molecular clock. The xml file produced by BEAUTi was manually edited to set the divergence between *V. pattersoni* and *V. nayarit* to a normal distribution with a mean age of 10.75 Ma and standard deviation of 2, producing soft upper and lower bounds set to the maximum (14 Ma) and minimum (7.5 Ma) ages for the separation of the Cape from mainland Mexico. The ulcd.mean parameter for the four gene partitions was given wide uniform distributions (0.02 upper bound and 0.002 lower bound for the two mtDNA genes; 0.01 and 0.0001 for the two nuclear genes) to allow rates across the species tree to be estimated by constraints placed by the Baja calibration and not by rates placed on individual genes. Analyses were run for 200 million generations, with samples retained every 1000 generations. Results were displayed in TRACER to confirm acceptable mixing and likelihood stationarity, appropriate burn-in, and adequate effective sample sizes. The first 10% of generations were discarded as burn-in, and parameter estimates summarized on the maximum clade credibility tree using TreeAnnotator v1.6.2 [49]. This burn-in and visualization procedure was repeated for each of the three gene trees co-estimated by *BEAST.

A relaxed Bayesian molecular clock framework implemented in BEAST was used in addition to estimate divergences from the complete mtDNA dataset. As with the mtDNA tree, the molecular clock was calibrated based on the split of the Cape region of Baja California from mainland Mexico. *Vaejovis pattersoni* and *V. nayarit* were grouped together as monophyletic, and the node representing the split of these two taxa was given a normal distribution with a mean age of 10.75 Ma and standard deviation of 2. Analyses were conducted for 40 million generations, with samples retained every 1000 generations, using a Yule tree prior. TRACER was used to confirm acceptable mixing and stationarity, appropriate burn-in, and adequate effective sample sizes. After discarding the first 4 million generations (10%) as burn-in, the parameter values of the samples from the posterior distribution were summarized on the maximum clade credibility tree using TreeAnnotator.

### Diversification rate analysis

Temporal shifts in diversification rates were analyzed using Maximum Likelihood-based diversification-rate analysis [50], using divergence dates estimated from the multilocus dataset with *BEAST and from the mtDNA dataset with BEAST. The fit of different birth–death models implementing two constant rates (pure-birth and birth–death) and three variable rates (exponential and logistic density-dependent, and two-rate pure-birth) was computed with LASER v2.3 [51]. Model fit was measured using AIC scores. The significance of the change in AIC scores (ΔAICrc) between the best rate-constant and best rate-variable model was determined by creating a null distribution for ΔAICrc. This was accomplished by simulating 1000 trees using yuleSim in LASER with the same number of nodes and same speciation rate estimated under the pure-birth model. Log likelihood and AIC values were calculated for three models (SPVAR, EXVAR, and BOTHVAR; described in [52]) that permit differential extinction and speciation rates. In addition, lineage-through-time plots were generated to visualize the pattern of accumulation of log-lineages over time. Another set of analyses were performed on the mtDNA-only divergence date estimates from BEAST to assess the influence of additional genetic structure present within lineages that may have been excluded from the diversification rate analyses above. Eight additional divergences that occurred within six of the inferred geographical lineages (see Fig. S1) were included for this dataset.

## Results

### Genetic Data

Sequences were deposited in GenBank (JX909361–JX909616). The mtDNA genes contained 418 parsimony-informative sites (283/854 bp, COI; 135/404 bp, 16S) in the full dataset and 413 sites in the reduced species tree dataset (279, COI; 134, 16S). The nuclear genes contained fewer parsimony-informative sites (30/526 bp, 28S; 128/319, ITS2). No significant conflict was found between the mitochondrial and nuclear genes (*P* = 0.8). GTR+I+G models of sequence evolution were selected for the CO1 and 16S partitions in the mtDNA dataset. In the species tree datasets, GTR+I+G was selected for CO1 and 16S, GTR+I for 28S, and GTR+G for ITS2.

### Phylogeographical analyses

Monophyly of the *vorhiesi* group was strongly supported in the mtDNA tree (Fig. 2). Strong phylogeographical signal was evident, but basal relationships were poorly resolved. Twenty-seven geographically cohesive lineages (including singletons, henceforth referred to as ‘lineages’ for convenience) were inferred from the mtDNA tree. Eleven of these lineages represented the species *V. bandido*, *V. cashi*, *V. deboerae*, *V. electrum*, *V. feti*, *V. halli*, *V. jonesi*, *V. lapidicola*, *V. paysonensis*, *V. tenuipalpus*, and *V. vorhiesi* and are referred to hereafter by their species names. The remaining 16 lineages consisted of samples from single mountain ranges with two exceptions. The exceptions were one lineage, referred to as ‘Mogollon’, that included samples from several mountains, and one lineage with samples from the adjacent Florida and Tres Hermanas mountains (Fig. 1).

### Species tree and divergence date estimates

**Figure 3:**
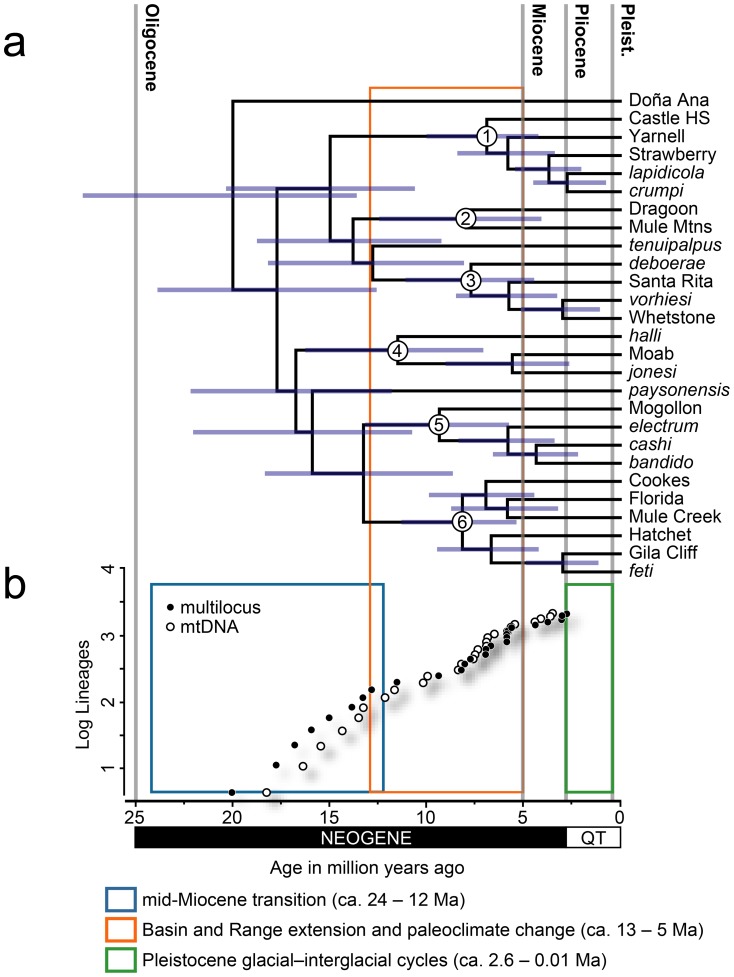
Tempo of diversification for scorpions in the *Vaejovis vorhiesi* group. (a) Time-calibrated species tree reconstructed using multilocus data. Numbers denote major geographical clades. These clades are mapped across the landscape in Figure 4. Bars indicate 95% highest posterior densities. Divergence times and posterior probability support values are provided in Figure S2. (b) Lineage through time plots derived from mtDNA and multilocus estimates of divergence dates. Birth–death likelihood analyses suggest a rate-variable density-dependent rate of diversification though time for both datasets. Approximate timing of major geological and climatic events that changed the landscape of southwestern North American are delineated, with time shown in millions of years (Ma). Pleist. = Pleistocene, QT = Quaternary.

The species tree reconstruction (Fig. 3) revealed relatively deep structure with long terminal branches similar to the mtDNA gene tree used for phylogeographical inference (Fig. 2). The three gene trees each revealed relatively heterogeneous tree topologies (Fig. S1). Six geographically cohesive groups of lineages were consistently inferred, suggesting that the species tree was not heavily biased by the mtDNA data. Differences between the species tree and mtDNA tree were confined to weakly supported nodes (see Fig. S2 for species tree posterior probability support values), resulting in topological rearrangements towards the base of the trees. The phylogenetic positions of the *paysonensis* and *tenuipalpus* lineages, in particular, differed among the topologies.

**Figure 4:**
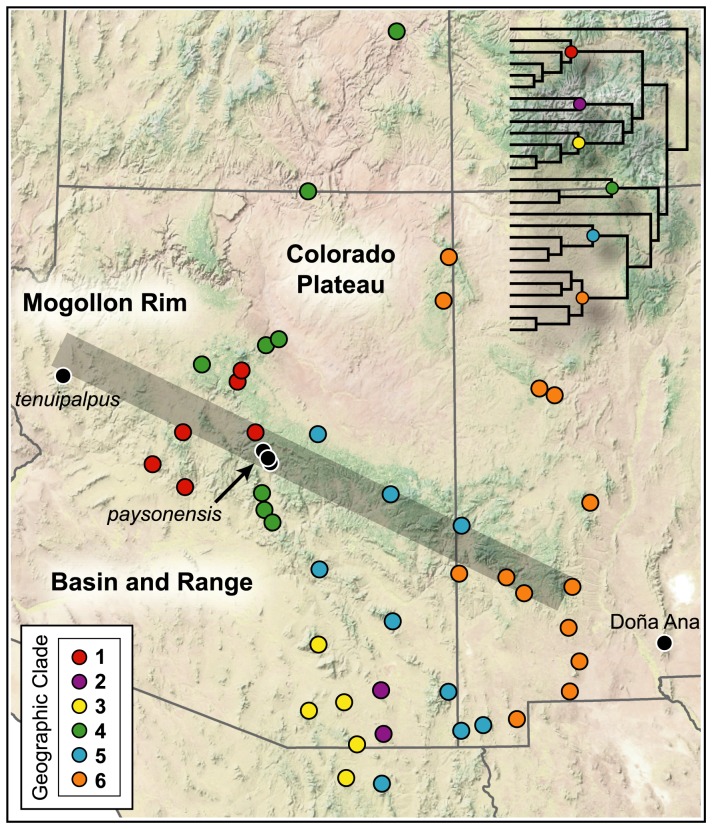
Geography of diversification among scorpions in the *Vaejovis vorhiesi* group. Color-coded dots, representing each of the six major geographical clades, are shown on the multilocus species tree (see Figure 3). Three lineages not within inferred major clades are labeled on the map. Sample names for each locality are provided in Figure 1. Gray bar indicates the approximate location of the Mogollon Rim.

Six geographically cohesive groups of lineages (referred to herein as ‘clades’; Clades 1–6, Figs. 3 and 4) and three lineages (*paysonensis*, *tenuipalpus*, and Doña Ana) formed the framework of the species tree. However, only the relationship between two clades (Clades 5 and 6) was strongly supported (Fig. S2). The geographical distribution of five of the six clades (Clades 1 and 4–6) and one of the two individual lineages (*tenuipalpus*) were anchored in the transition zone between the Colorado Plateau and the Basin and Range geological provinces, along the Mogollon Rim (Fig. 4). Two clades of Madrean ‘sky island’ endemics (Clades 2 and 3) were confined to the Basin and Range province. The *tenuipalpus* and Doña Ana lineages were respectively situated at the eastern and western extremes of the distribution of the *vorhiesi* group.

Divergence date estimates inferred from the Cape-calibrated multilocus and mtDNA datasets using *BEAST and BEAST were similar (Fig. S2). Mean divergence dates were generally within 2 Ma, whereas posterior credibility intervals were slightly narrower in the multilocus analyses. Divergence dates estimated across the species tree (Fig. 3 and Fig. S2) suggested that early diversification in the *vorhiesi* group began around 20 Ma. Although 95% posterior credible intervals were wide, most divergences estimated within the *vorhiesi* group were within the Neogene period from 25 to 2.6 Ma. Only five of the 26 inferred divergences incorporated the Pleistocene within credible intervals, and all mean divergence date estimates pre-dated the Pleistocene.

### Tempo of diversification

Birth–death likelihood analyses of lineage diversification rates rejected the null hypothesis of rate-constancy for both datasets (*P* = 0.003, multilocus; *P*<0.001, mtDNA-only). The rate-variable model that best fit the datasets was the logistic density-dependent (DDL) model. Under this model, diversification rate in the *vorhiesi* group gradually decreased through time, with diversification rate estimated at 0.276 (multilocus *BEAST) or 0.370 (mtDNA-only BEAST) divergences per million years (Fig. 3). Models considering variable rates of extinction and speciation did not provide a better fit to the datasets. Similar results (rejection of the null hypothesis, *P* = 0.01; DDL best rate-variable model) were obtained when more recent divergences within lineages were accounted for (Fig. S2).

## Discussion

### Lineage diversification across southwestern North America

The time-calibrated phylogenetic hypothesis presented here suggests that scorpions in the *vorhiesi* group have had a long history in the highlands of southwestern North America. Results reveal a striking pattern of lineage diversification almost entirely confined within the bounds of the Neogene period (Fig. 3). Based on mean estimates, all 26 inferred cladogenetic events probably occurred prior to the Pleistocene epoch. Only five divergences incorporated the Pleistocene within credible intervals (Fig. S2).

Geological processes that cumulatively acted to separate the Basin and Range province from the Colorado Plateau occurred during a period known as the mid-Miocene transition [53], [54]. This episode of geological activity, 24–12 Ma, probably created the landscape in which the *vorhiesi* group originated. The transformation of an originally more continuous landscape to one of isolated mountain ranges associated with Basin and Range extension may have provided opportunities for rapid early diversification. The largely unsupported basal divergences in the species tree that occurred around 18–13 Ma (Fig. S2) are consistent with a rapid series of divergences. It is difficult to resolve these specific relationships with the current dataset, and whether this represents a case of simultaneous divergence from a common ancestor (i.e., hard polytomy) or estimation uncertainty (i.e., soft polytomy) remains unknown. Regardless, it is clear that the early divergences within these scorpions occurred in rapid succession over a narrow window of time (Fig. S2).

Following early diversification in the *vorhiesi* group, potentially linked with the mid-Miocene transition, the development of regional clades between about 12–7 Ma (Fig. 3) may have initiated in response to continued landscape deformation coincident with paleoclimatical change [55]. Basin and Range extension in southern Arizona may have spread rapidly northeastward across southern Arizona into the Rio Grande rift of southern New Mexico between about 13–9 Ma [56], [57], [58]. This development was synchronous with climatic change in the area that was punctuated by a dramatic shift in atmospheric conditions at 7–5 Ma [55], [59]. The distributions of four of the six inferred regional clades (Clades 2, 3, 5, and 6) and one lineage (Doña Ana) extend across southern Arizona and New Mexico (Fig. 4). The two remaining clades to the north (Clades 1 and 4) are centered along the Mogollon Rim. Late Miocene formation of localized volcanic fields across the Mogollon Rim [60] and tilting and faulting, around 13–6 Ma during the extension of the Basin and Range [54], may have contributed to diversification at the base of these two clades.

### Fine-scale ancient endemism

Mountainous regions are known to generally harbor greater species endemism than adjacent lowland areas [61], [62], [63]. Much of this endemism resulted from Quaternary climatic oscillations driving fragmentation and isolation of highland habitats (e.g., [23]). Scorpions in the *vorhiesi* group display remarkable patterns of fine-scale endemism that appear to have developed much earlier than the Pleistocene. This is unsurprising given their stenotopic habitat requirements [21]. Diversification within the *vorhiesi* group was probably driven by allopatry in isolated rocky highland habitats: ephemeral woodland corridors may have connected highland biota during the Pleistocene but lacked suitable humid, rocky habitats required by these scorpions for dispersal between adjacent mountain ranges. The persistence of separate lineages at small spatial scales suggests that a combination of ecological stenotopy and limited vagility make the *vorhiesi* group particularly valuable as indicators of geomorphological evolution in southwestern North America, as observed in other scorpion taxa elsewhere in the world [9], [11].

### Tempo of diversification

Diversification rate in the *vorhiesi* group appears to have decreased slowly through time (Fig. 3). Given the restricted distributions and stenotopic requirements of *vorhiesi* group scorpions for insular montane habitats, this finding is consistent with studies suggesting that diversification rate slows as ecological opportunities diminish [52], [64], [65], [66], and that declining diversification rates may be caused by the saturation of geographical space [67]. The decreased diversification rate of these scorpions is probably related to the dynamic geological history of southwestern North America previously outlined, which appears sufficient to have formed new habitats and dispersal routes, and to have isolated older habitats throughout the Neogene. Divergence times in the *vorhiesi* group suggest that these highland habitats were occupied and persisted throughout the Neogene and that, by the onset of Quaternary, few unoccupied habitats would have been available. Quaternary climate change, while dramatically shifting vegetation communities across southwestern North America [68], may have had little impact on diversification in these stenotopic scorpions.

### Montane scorpions as windows to pre-Quaternary history and orogeny

A range of genetic divergences across a Neogene temporal continuum, combined with fine-scale endemism, suggests that montane scorpions offer the potential to assess correlations between Earth history and biotic diversification. Millions of years of dynamic change across a heterogeneous landscape chiseled a genetic footprint in scorpions of the *vorhiesi* group that has not eroded through potential Pleistocene habitat connectivity. These scorpions can thus provide unique insight into the impact of pre-Quaternary events on the generation of biodiversity. Perhaps more importantly, these events can be inferred at a relatively fine geographical scale.

Agreement on a cohesive geohistorical framework for mountain formation across southwestern North America remains elusive despite decades of research [69]. Ecologically specialized ‘living fossils’, like the scorpions of the *vorhiesi* group, represent model organisms for tracking orogeny, as evidenced in the present study by the amount of genetic structure observed across the relatively small distances between adjacent mountain ranges. Other stenotopic scorpions may offer similar insights regarding mountain formation and the generation of diversity on montane islands.

## Supporting Information

Figure S1 **Three gene trees for scorpions in the** ***Vaejovis vorhiesi*** **group embedded within the shared species tree.** Strongly supported nodes (≥0.95 posterior probability support) are indicated with black dots.
(PDF)

Figure S2 **Chronograms with estimated divergence times (in millions of years, Ma) for scorpions in the** ***Vaejovis vorhiesi*** **group.** Estimates are shown for multilocus species tree (top) and mtDNA gene tree (bottom) analyses. Posterior probability support values for nodes are indicated by coded dots explained in the figure legend. Nodes that received <0.50 support are not indicated with dots. Means and 95% highest posterior densities (in brackets) are shown for each node. Asterisks indicate eight additional divergences included in diversification rate analyses (see Methods and Materials). QT = Quaternary.
(PDF)

Table S1 **Scorpion-specific mtDNA primers used in study on the** ***Vaejovis vorhiesi*** **group.**
(DOC)

## References

[1] Morrone JJ (2009) Evolutionary Biogeography: An Integrative Approach with Case Studies. New York: Columbia University Press. 304 p.

[2] Lomolino MV, Riddle BR, Whittaker RJ, Brown JH (2010) Biogeography, 4^th^ edition. Sunderland, Massachusetts: Sinauer Associates, Inc. 764 p.

[3] Cody S, Richardson JE, Rull V, Ellis C, Pennington RT (2010) The great American biotic interchange revisited. Ecography 33: 326–332.

[4] Smith BT, Klicka J (2010) The profound influence of the Late Pliocene Panamanian uplift on the exchange, diversification, and distribution of New World Birds. Ecography 33: 333–342.

[5] Wiens JJ (2011) The niche, large-scale biogeography, and species interactions. Philos Trans R Soc Lond 366: 2336–2350. 21768150 10.1098/rstb.2011.0059 PMC3130432

[6] Avise J (2000) Phylogeography: the History and Formation of Species. Harvard University Press, Cambridge, MA. 447 p.

[7] Knowles LL (2009) Statistical phylogeography. Annu Rev Ecol Syst 40: 593–612.

[8] Hickerson MJ, Carstens BC, Cavender-Bares J, Crandall KA, Graham CH, et al (2010) Phylogeography's past, present, and future: 10 years after Avise 2000. Mol Phylogenet Evol 54: 291–301. 19755165 10.1016/j.ympev.2009.09.016

[9] Prendini L (2005) Scorpion diversity and distribution in southern Africa: Pattern and process. In: Huber BA, Sinclair BJ, Lampe K-H, editors. African Biodiversity: Molecules, Organisms, Ecosystems. New York Springer Verlag. pp 25–68.

[10] Coddington JA, Giribet G, Harvey MS, Prendini L, Walter DE (2004) Arachnida. In: Cracraft J, Donoghue M.Oxford, editors. Assembling the Tree of Life. Oxford University Press. pp 296–318.

[11] Prendini L (2001) Substratum specialization and speciation in southern African scorpions: the Effect Hypothesis revisited. In: Fet V, Selden PA, editors. In Memoriam Gary A. Polis, British Arachnological Society. Burnham Beeches, Bucks. pp 113–138.

[12] Gantenbein B, Largiadèr CR (2003) The phylogeographic importance of the Strait of Gibraltar as a gene flow barrier in terrestrial arthropods: A case study with the scorpion *Buthus occitanus* as model organism. Mol Phylogenet Evol 28: 119–130. 12801475 10.1016/s1055-7903(03)00031-9

[13] Parmakelis A, Stathi I, Spanos L, Louis C, Mylonas M (2006) Phylogeography of *Iurus dufoureius* (Brulle, 1832) (Scorpiones, Iuridae). J Biogeogr 33: 251–260.

[14] Parmakelis A, Stathi I, Chatzaki M, Simaiakis S, Spanos L, et al (2006) Evolution of *Mesobuthus gibbosus* (Brulle, 1832) (Scorpiones : Buthidae) in the northeastern Mediterranean region. Mol Ecol 15: 2883–2894. 16911208 10.1111/j.1365-294X.2006.02982.x

[15] Borges A, Bermingham E, Herrera N, Alfonzo M, Sanjur O (2010) Molecular systematics of the neotropical scorpion genus *Tityus* (Buthidae): The historical biogeography and venom antigenis diversity of toxic Venezuela species. Toxicon 55: 436–454. 19799925 10.1016/j.toxicon.2009.09.011

[16] Sousa P, Froufe E, Alves PC, Harris DJ (2010) Genetic diversity within scorpions of the genus *Buthus* from the Iberian Peninsula: mitochondrial DNA sequence data indicate additional distinct cryptic lineages. J Arachnol 38: 206–211.

[17] Lourenço WR, Sissom WD (2000) Scorpiones. In: Bousquets JL, González Soriano E, Papavero, editors. Biodiversidad, Taxonomía y Biogeographía de Artrópodos de México. Hacia una Síntesis de su Concimiento. Volume 2. Ciudad de México: Universidad Nacional Autónoma de México. pp 115–135.

[18] Sissom WD, Hendrixson BE (2005) Scorpion biodiversity and patterns of endemism in northern Mexico. In: Cartron J-LE, Ceballos G, editors. Biodiversity, Ecosystems, and Conservation in northern Mexico. Oxford: Oxford University Press. pp 122–137.

[19] Bryson RW, García-Vázquez UO, Riddle BR (2011) Relative roles of Neogene vicariance and Quaternary climate change on the historical diversification of bunchgrass lizards (*Sceloporus scalaris* group) in Mexico. Mol Phylogenet Evol 62: 447–457. 22075377 10.1016/j.ympev.2011.10.014

[20] Bryson RW, Murphy RW, Graham MR, Lathrop A, Lazcano-Villareal D (2011) Historical diversification of the *Crotalus intermedius* group and Pleistocene pine-oak woodland connections between Mexico's Sierra Madre Occidental and Sierra Madre Oriental. J Biogeogr 38: 2299–2310.

[21] Sissom WD, Hughes GB, Bryson RW, Prendini L (2012) The *vorhiesi* group of *Vaejovis* C.L. Koch, 1836 (Scorpiones: Vaejovidae) in Arizona, with description of a new species from the Hualapai Mountains. Am Mus Novit 3742: 1–19.

[22] Graham MR, Ayrey RF, Bryson RW (2012) Multivariate methods support the distinction of a new highland *Vaejovis* (Scorpiones: Vaejovidae) from the Sierra de los Ajos, Mexico. J Arachnol 40: 281–290.

[23] McCormack JE, Bowen BS, Smith TB (2008) Integrating paleoecology and genetics of bird populations in two sky island archipelagos. BMC Biol 6: 28. 18588695 10.1186/1741-7007-6-28 PMC2474579

[24] Knowles LL (2001) Genealogical portraits of speciation in montane grasshoppers (genus *Melanoplus*) from the sky islands of the Rocky Mountains. Proc R Soc B 268: 319–324. 10.1098/rspb.2000.1364 PMC1088609 11217904

[25] Ayoub NA, Riechert SE (2004) Molecular evidence for Pleistocene glacial cycles driving diversification of a North American desert spider, *Agelenopsisaperta* . Mol Ecol 13: 3453–3465. 15488003 10.1111/j.1365-294X.2004.02335.x

[26] Wood DA, Vandergast AG, Lemos-Espinal JA, Fisher RN, Holycross AT (2011) Refugial isolation and divergence in the Narrowheaded Gartersnake species complex (*Thamnophis rufipunctatus*) as revealed by multilocus DNA sequence data. Mol Ecol 20: 3856–3878. 21851436 10.1111/j.1365-294X.2011.05211.x

[27] Stockwell SA (1989) Revision of the Phylogeny and Higher Classification of Scorpions (Chelicerata). PhD Dissertation. University of California, Berkeley. 319 p.

[28] Moulton MJ, Song H, Whiting MF (2010) Assessing the effects of primer specificity on eliminating numt coamplification in DNA barcoding: a case study from Orthoptera (Arthropoda: Insecta). Mol Ecol Res 10: 615–627. 10.1111/j.1755-0998.2009.02823.x 21565066

[29] Bertheau C, Schuler H, Krumböck S, Arthofer W, Stauffer C (2011) Hit or miss in phylogeographic analyses: the case of the cryptic NUMTs. Mol Ecol Res 11: 1056–1059. 10.1111/j.1755-0998.2011.03050.x 21791032

[30] Tully T, D'Haese C, Richard M, Ferrière R (2006) Two major evolutionary lineages revealed by molecular phylogeny in the parthenogenetic collembolan *Folsomia candida* . Pedobiologia 50: 95–104.

[31] Ji Y-J, Zhang D-X, He L-J (2003) Evolutionary conservation and versatility of a new set of primers for amplifying the ribosomal internal transcribed spacer regions in insects and other invertebrates. Mol Ecol Notes 3: 581–585.

[32] Prendini L, Crowe TM, Wheeler WC (2003) Systematics and biogeography of the family Scorpionidae Latreille, with a discussion of phylogenetic methods. Invert Syst 17: 185–259.

[33] Prendini L, Weygoldt P, Wheeler WC (2005) Systematics of the *Damon variegatus* group of African whip spiders (Chelicerata: Amblypygi): evidence from behaviour, morphology and DNA. Org Divers Evol 5: 203–236.

[34] Katoh K, Toh H (2008) Improved accuracy of multiple ncRNA alignment by incorporating structural information into a MAFFT-based framework. BMC Bioinformatics 9: 212. 18439255 10.1186/1471-2105-9-212 PMC2387179

[35] Katoh K, Misawa K, Kuma K, Miyata T (2002) MAFFT: a novel method for rapid multiple sequence alignment based on fast Fourier transform. Nucleic Acids Res 30: 3059–3066. 12136088 10.1093/nar/gkf436 PMC135756

[36] Stephens M, Donnelly P (2003) A comparison of Bayesian methods for haplotype reconstruction from population genotype data. Am J Hum Genet 73: 1162–1169. 14574645 10.1086/379378 PMC1180495

[37] Swofford DL (2002) PAUP*: Phylogenetic Analysis Using Parsimony (*and Other Methods), Version 4.0b10. Sinauer, Sunderland, Massachusetts.

[38] Ronquist F, Huelsenbeck JP (2009) MRBAYES 3: Bayesian phylogenetic inference under mixed models. Bioinformatics 19: 1572–1574. 10.1093/bioinformatics/btg180 12912839

[39] Avise JC, Walker D (1998) Pleistocene phylogeographic effects on avian populations and the speciation process. Proc R Soc B 265: 457–463. 10.1098/rspb.1998.0317 PMC1688909 9569664

[40] Avise JC, Walker D, Johns GC (1998) Speciation durations and Pleistocene effects on vertebrate phylogeography. Proc R Soc B 265: 1707–1712. 10.1098/rspb.1998.0492 PMC1689361 9787467

[41] Rissler LJ, Apodaca JJ (2007) Adding more ecology into species delimitation: ecological niche models and phylogeography help define cryptic species in the black salamander (*Aneides flavipunctatus*). Syst Biol 56: 924–942. 18066928 10.1080/10635150701703063

[42] Kass R, Raftery A (1995) Bayes factors. J Am Statist Assoc 90: 773–795.

[43] Nylander JAA (2004) MRMODELTEST v2. Program distributed by the author. Evolutionary Biology Centre, Uppsala University, Uppsala.

[44] Rambaut A, Drummond AJ (2012) Tracer v1.5. Available: http://beast.bio.ed.ac.uk/Tracer. Accessed 2011 Sep 12.

[45] Heled J, Drummond A (2010) Bayesian inference of species trees from multilocus data. Mol Biol Evol 27: 570–580. 19906793 10.1093/molbev/msp274 PMC2822290

[46] Ferrari L (1995) Miocene shearing along the northern boundary of the Jalisco block and the opening of the southern Gulf of California. Geology 23: 751–754.

[47] Oskin M, Stock J (2003) Marine incursions synchronous with plate-boundary localization in the Gulf of California. Geology 31: 23–26.

[48] Mulcahy DG, Macey JR (2009) Vicariance and dispersal form a ring distribution in nightsnakes around the Gulf of California. Mol Phylogenet Evol 53: 537–546. 19501659 10.1016/j.ympev.2009.05.037

[49] Drummond AJ, Rambaut A (2007) BEAST: Bayesian evolutionary analysis by sampling trees. BMC Evol Biol 7: 214. 17996036 10.1186/1471-2148-7-214 PMC2247476

[50] Rabosky DL (2006) Likelihood methods for inferring temporal shifts in diversification rates. Evolution 60: 1152–1164. 16892966

[51] Rabosky DL (2006) LASER: a maximum likelihood toolkit for detecting temporal shifts in diversification rates from molecular phylogenies. Evol Bioinformatics Online 2: 257–260. PMC2674670 19455217

[52] Rabosky DL, Lovette IJ (2008) Density dependent diversification in North American wood warblers. Proc R Soc B 275: 2363–2371. 10.1098/rspb.2008.0630 PMC2603228 18611849

[53] Shafiqullah M, Damon PE, Lynch DJ, Reynolds SJ, Rehrig WA, et al.. (1980) K-Ar geochronology and geologic history of southwestern Arizona and adjacent area. In: Jenney J, Stone C, editors. Studies in Western Arizona: Arizona Geological Society Digest, Vol. XII. Arizona Geological Society: Tucson. pp 201–260.

[54] Brand P, Stump E (2011) Tertiary Extension and Fault Block Rotation in the Transition Zone, Cedar Mountains Area, Arizona v.1.1. Arizona Geological Survey, Tucson.

[55] Retallack GJ (2001) Cenozoic expansion of grasslands and climatic cooling. J Geol 109: 407–426.

[56] Seager WR, Shafiqullah M, Hawley JW, Marvin RF (1984) New K–Ar dates from basalts and the evolution of the southern Rio Grande rift. Geol Soc Am Bull 95: 87–99.

[57] Menges CM, Pearthree PA (1989) Late Cenozoic tectonism in Arizona and its impact on regional landscape evolution. In: Jenney JP, Reynolds SJ, editors. Geologic Evolution of Arizona. Tucson: Arizona Geological Society. pp 649–680.

[58] Henry CD, Aranda-Gomez J (2000) Plate interactions control middle–late Miocene, proto-Gulf and Basin and Range extension in the southern Basin and Range. Tectonophysics 318: 1–26.

[59] Retallack GJ (1997) Neogene expansion of the North American Prairie. Palaios 12: 380–390.

[60] Sabels BE (1962) Mogollon Rim volcanism and geochronology. In: Weber RH, Peirce HW, editors. Guidebook of the Mogollon Rim Region, East-central Arizona, New Mexico Geological Society 13th Field Conference. Socorro: New Mexico Geological Society. pp 100–107.

[61] Wiens JJ, Parra-Olea G, Garcia-Paris M, Wake DB (2007) Phylogenetic history underlies elevational patterns of biodiversity in tropical salamanders. Proc R Soc B 274: 919–928. 10.1098/rspb.2006.0301 PMC2141676 17284409

[62] Thomas GH, Orme CDL, Davies RG, Olson VA, Bennett PM, et al (2008) Regional variation in the historical components of global avian species richness. Global Ecol Biogeogr 17: 340–351.

[63] Badgley C (2010) Tectonics, topography, and mammalian diversity. Ecography 33: 220–231.

[64] McPeek MA (2008) The ecological dynamics of clade diversification and community assembly. Am Nat 172: E270–E284. 18851684 10.1086/593137

[65] Kozak KH, Weisrock DW, Larson A (2006) Rapid lineage accumulation in a non-adaptive radiation: phylogenetic analysis of diversification rates in eastern North American woodland salamanders (Plethodontidae: *Plethodon*). Proc R Soc B 273: 539–546. 10.1098/rspb.2005.3326 PMC1560065 16537124

[66] Burbrink FT, Pyron RA (2010) How does ecological opportunity influence rates of speciation, extinction, and morphological diversification in New World ratsnakes (tribe Lampropeltini)? Evolution 64: 934–943. 19895554 10.1111/j.1558-5646.2009.00888.x

[67] Pigot AL, Phillimore AB, Owens IPF, Orme CDL (2010) The shape and temporal dynamics of phylogenetic trees arising from geographic speciation. Syst Biol 59: 660–673. 20952757 10.1093/sysbio/syq058

[68] Van Devender TR (1990) Late Quaternary vegetation and climate of the Sonoran Desert, United States and Mexico. In: Betancourt JL, Van Devender TR, Martin PS, editors. Packrat Middens. The Last 40,000 Years of Biotic Change. Tucson: University of Arizona Press. pp 134–165.

[69] Wilson JS, Pitts JP (2010) Illuminating the lack of consensus among descriptions of earth history data in the North American deserts: a resource for biologists. Prog Phys Geog 34: 419–441.

