# Supplementary figures and images for: As Old as the Hills: Montane Scorpions in Southwestern North America Reveal Ancient Associations between Biotic Diversification and Landscape History
Source: PLoS One. 2013 Jan 9;8(1):e52822. doi: 10.1371/journal.pone.0052822 (PMC3541388; doi:10.1371/journal.pone.0052822)

mtDNA

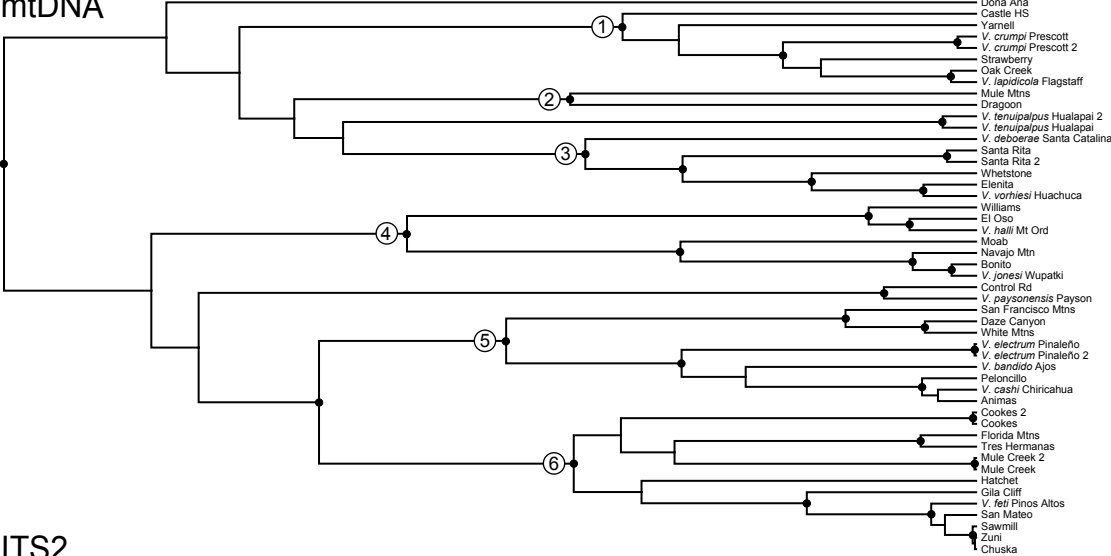

ITS2

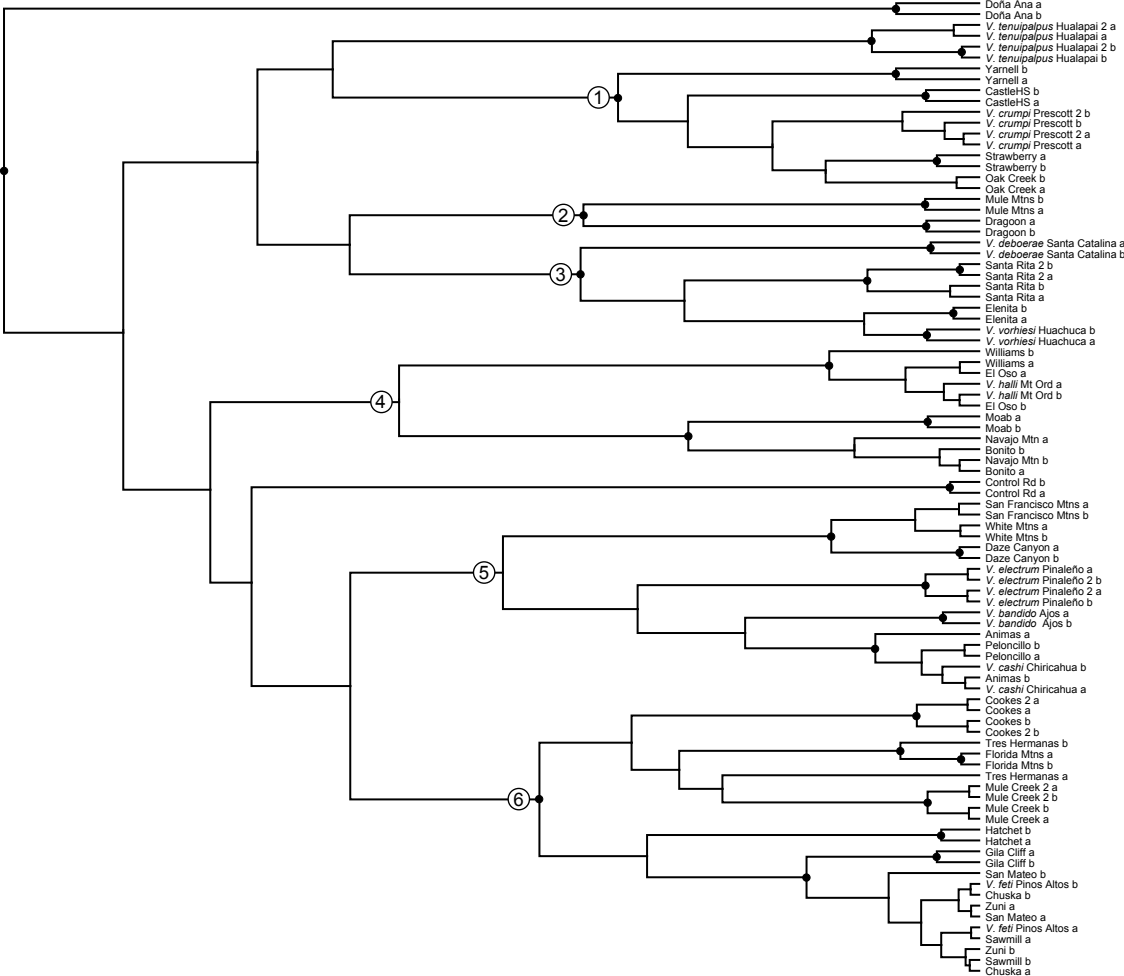

28S

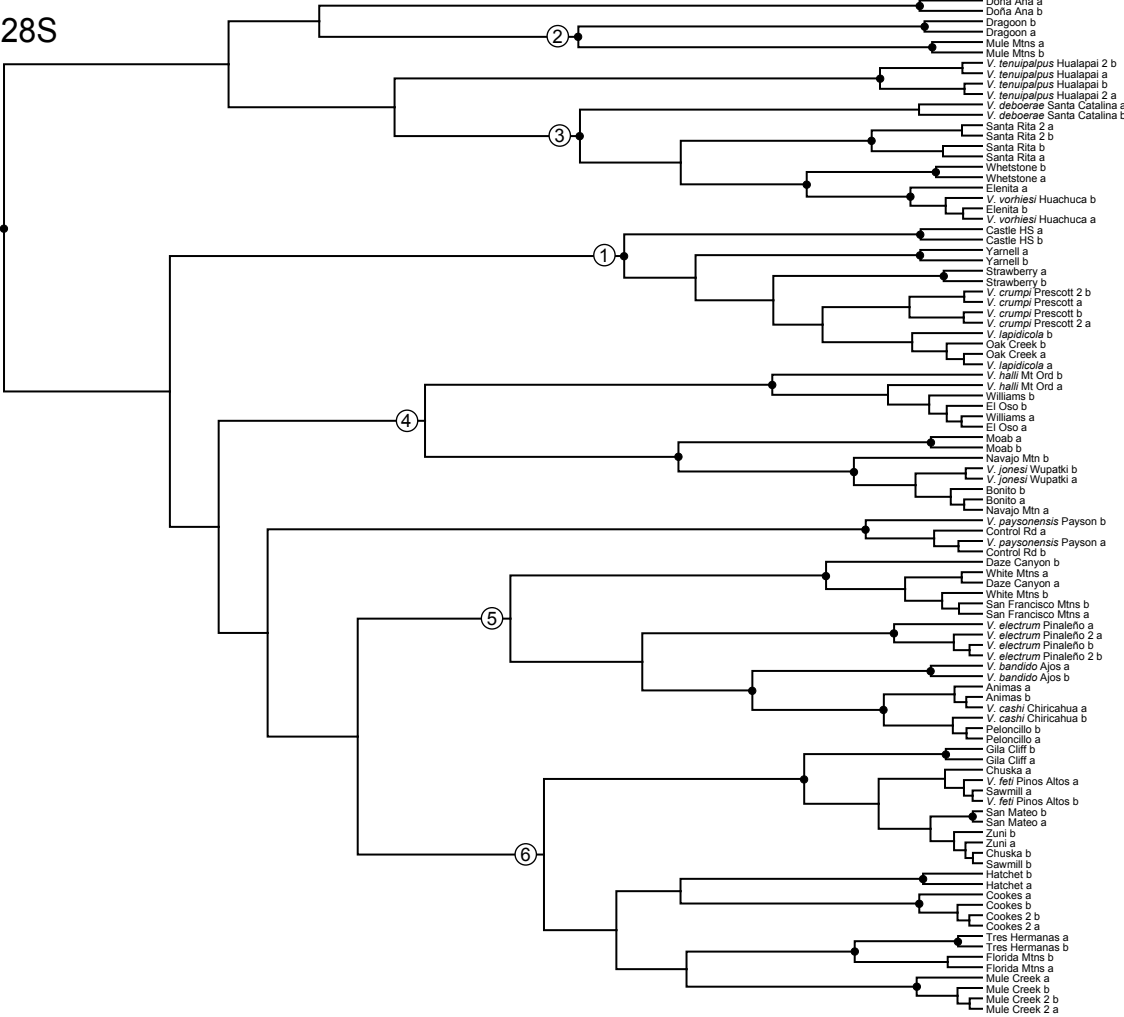

Supplement: Figure S1 — Three gene trees for scorpions in the Vaejovis vorhiesi group embedded within the shared species tree. Strongly supported nodes (≥0.95 posterior probability support) are indicated with black dots. (PDF) [file pone.0052822.s001.pdf]

Multilocus \*BEAST

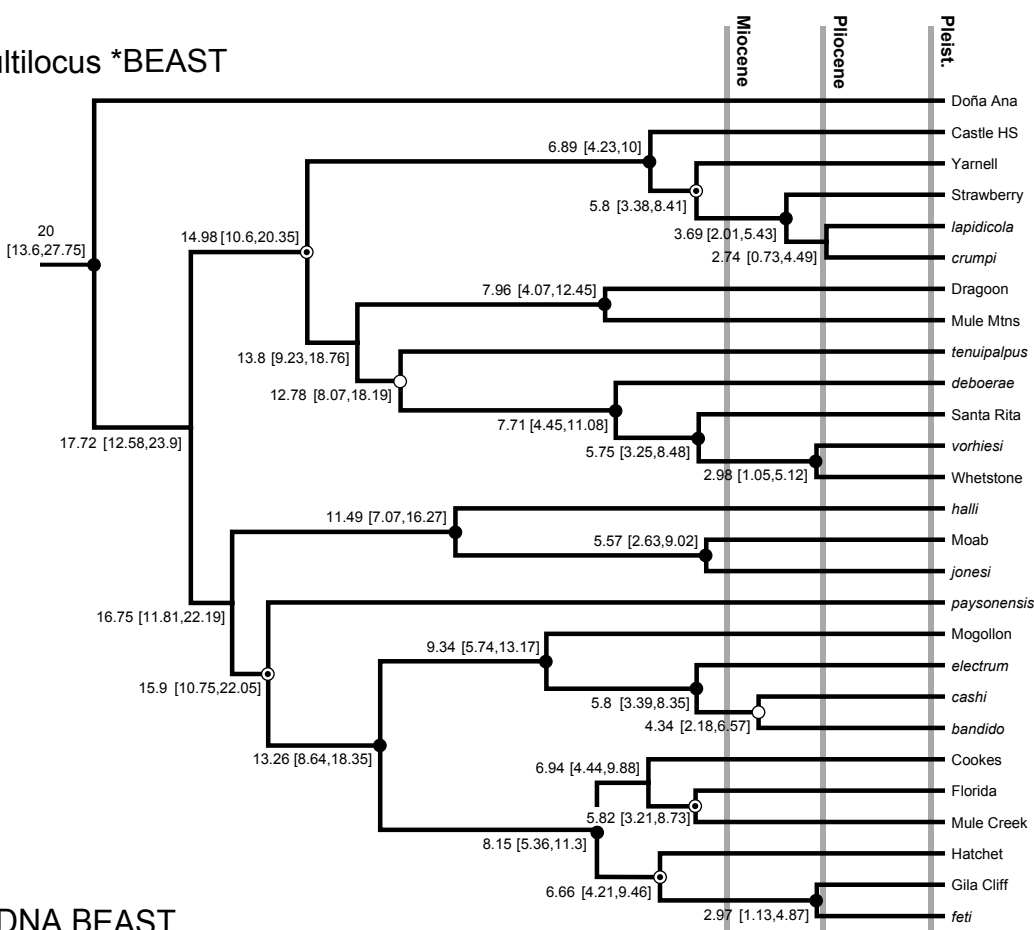

mtDNA BEAST

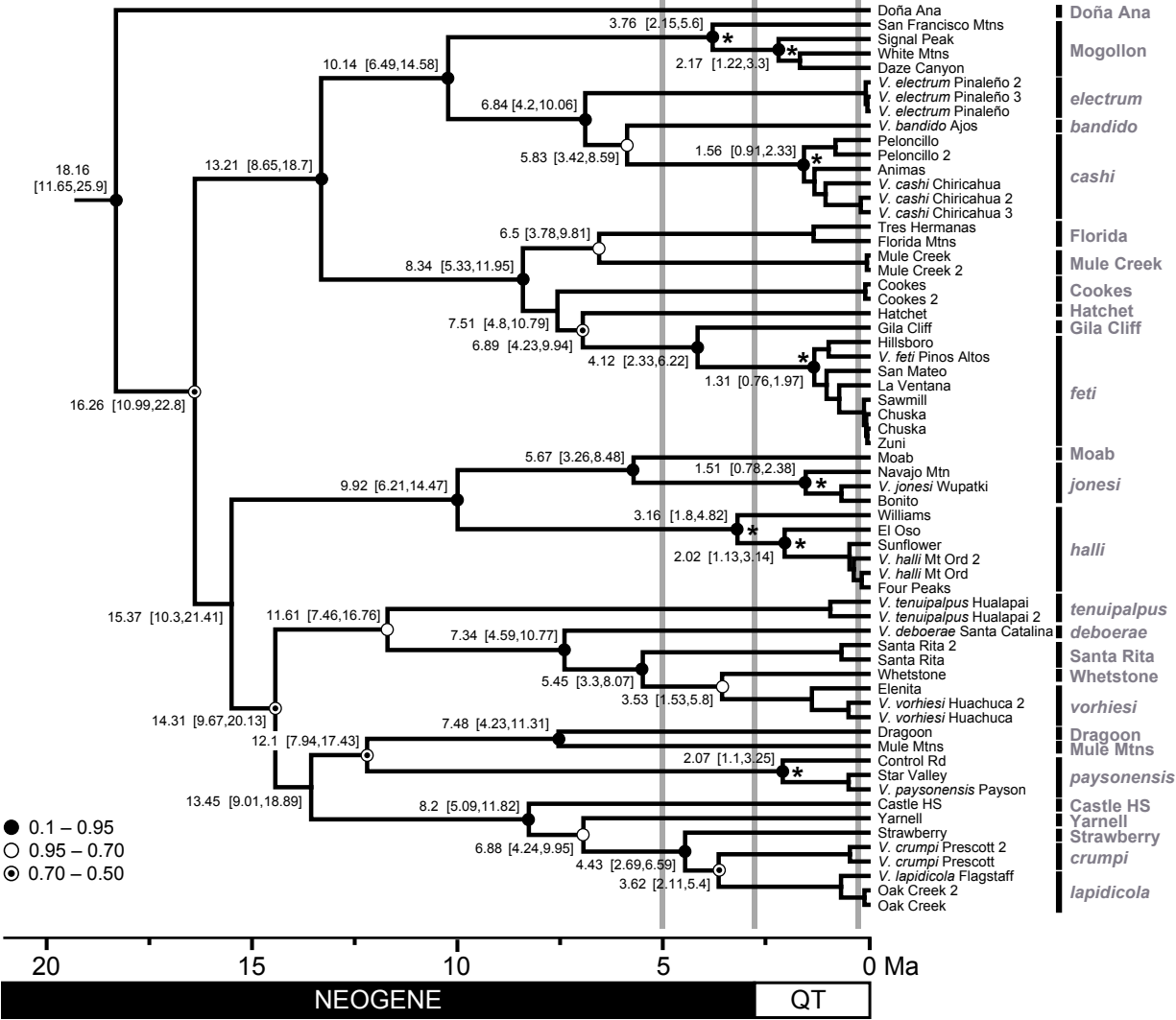

Supplement: Figure S2 — Chronograms with estimated divergence times (in millions of years, Ma) for scorpions in the Vaejovis vorhiesi group. Estimates are shown for multilocus species tree (top) and mtDNA gene tree (bottom) analyses. Posterior probability support values for nodes are indicated by coded dots explained in the figure legend. Nodes that received <0.50 support are not indicated with dots. Means and 95% highest posterior densities (in brackets) are shown for each node. Asterisks indicate eight additional divergences included in diversification rate analyses (see Methods and Materials). QT = Quaternary. (PDF) [file pone.0052822.s002.pdf]
