# Supplementary material for: As Old as the Hills: Montane Scorpions in Southwestern North America Reveal Ancient Associations between Biotic Diversification and Landscape History
Source: PLoS One. 2013 Jan 9;8(1):e52822. doi: 10.1371/journal.pone.0052822 (PMC3541388; doi:10.1371/journal.pone.0052822)
Supplement: Table S1 — Scorpion-specific mtDNA primers used in study on the Vaejovis vorhiesi group. (DOC) [file pone.0052822.s003.doc]

**Table S1. Scorpion-specific mtDNA primers used in study on the *Vaejovis vorhiesi* group.**

| Primer name | Sequence | Source |
| --- | --- | --- |
| CO1modF | 5'-ATCATAAGGATATTGGGACTATGT-3' | This study |
| LE1r | 5'-GTAGCAGCAGTAAARTARGCYCGAGTATC-3' | [1] |
| 16SmodF | 5'-CACCGRTTTGAACTCAGATCA-3' | This study |
| 40R | 5'-gtgcaaaggtagcataatca-3' | [2] |

**References**

1. Esposito LA (2011) Systematics and Biogeography of the New World Scorpion Genus *Centruroides* Marx, 1890 (Scorpiones: Buthidae)*.* PhD Dissertation. City University of New York. 322 p.
2. Gantenbein B, Fet V, Largiadèr CR, Scholl A (1999) First DNA phylogeny of *Euscorpius* Thorell, 1876 (Scorpiones, Euscorpiidae) and its bearing on taxonomy and biogeographic of this genus. Biogeographica 75: 49–65.
